# Validation, Content Validity, and Reliability of the Spanish SE-OAM Questionnaire: Assessing Nursing Self-Efficacy in Oral Anticoagulant Therapy Management

**DOI:** 10.3390/clinpract15060111

**Published:** 2025-06-16

**Authors:** Juan Ramón de-Moya-Romero, Raquel Valera-Lloris, Elena Chover-Sierra, Laura Fernández-Puerta, Alexis Caballero-Bonafé, Antonio Martínez-Sabater

**Affiliations:** 1 Valencia Clinic Hospital, 46010 Valencia, Spain; demoya_jua@gva.es (J.R.d.-M.-R.); valera_raq@gva.es (R.V.-L.); fernandez_laupue@gva.es (L.F.-P.); caballero_alebon@gva.es (A.C.-B.); antonio.martinez-sabater@uv.es (A.M.-S.); 2 Nursing Department, Faculty of Nursing and Podiatry, University of Valencia, 46010 Valencia, Spain; 3 Nursing Care and Education Research Group (GRIECE), Nursing Department, Faculty of Nursing and Podiatry, University of Valencia, 46010 Valencia, Spain; 4 Care Research Group (INCLIVA), Valencia Clinic Hospital, 46010 Valencia, Spain; 5 Internal Medicine Department, Hospital General Universitario, 46014 Valencia, Spain; 6 Nursing Department, Faculty of Health Sciences, European University of Valencia, 46010 Valencia, Spain

**Keywords:** nursing, oral anticoagulants management, self-efficacy, instrument development, instrument validation, psychometrics

## Abstract

**Background/Objectives:** Oral anticoagulant therapy (OAT) has been prescribed for over seventy years to prevent thromboembolic complications associated with various conditions. The emergence of direct-acting oral anticoagulants (DOACs) has reduced the use of vitamin K antagonists (VKAs), but specific clinical scenarios still necessitate VKAs. Nurses play a crucial role in managing OAT, and their self-efficacy is essential for optimal patient outcomes. This study aims to validate and adapt the Nursing Self-Efficacy for Oral Anticoagulant Therapy Management (SE-OAM) questionnaire to Spanish (SE-OAM-SV) to assess nurses’ self-efficacy in managing OAT. **Methods:** A methodological design was employed to develop the validity and reliability of the SE-OAM-SV. The process included translation and back-translation, expert review, and a pilot study. Content validity was analyzed using the content validity index (CVI), modified kappa coefficient, and Aiken’s V. A descriptive cross-sectional study was conducted with 100 nurses across Spain to test the SE-OAM-SV and identify comprehension issues. Internal consistency was assessed via Cronbach’s alpha. **Results:** The translation process highlighted some items requiring clarification, which were resolved through expert consultation. The SE-OAM-SV demonstrated adequate content validity with a global CVI of 0.86. The pilot study revealed an average participant age of 41.3 years and 17.3 years of professional experience. The SE-OAM-SV showed high internal consistency with a Cronbach’s alpha of 0.96. The average score of participants on the SE-OAM-SV was 56.8 points, indicating room for improvement in all aspects of the scale. **Conclusion**: The SE-OAM-SV is a reliable and valid tool for measuring nurses’ self-efficacy in managing OAT in Spanish-speaking communities. This tool can facilitate the development of educational programs and public policies to enhance nurses’ self-efficacy and improve patient outcomes. The availability of the SE-OAM-SV supports larger-scale studies and validation in other Spanish-speaking countries.

## 1. Introduction

Oral anticoagulant therapy (OAT) has been prescribed for over seventy years to prevent thromboembolic complications associated with various conditions, such as atrial fibrillation, venous thromboembolic disease, and cardiac valve replacement [1,2].

Currently, we can differentiate between two types of OAT: vitamin K antagonists (VKAs) and direct-acting oral anticoagulants (DOACs). Until DOACs emerged, the oral alternative for anticoagulating patients was through VKAs. VKAs are characterized by a narrow therapeutic range that requires frequent monitoring, leading to dose adjustments with significant intra- and interpatient variability [2,3]. DOACs, which do not require routine monitoring, are administered at a fixed dose and have fewer interactions with other medications and foods; the use of VKAs has significantly declined. However, there are clinical scenarios where DOACs are contraindicated, such as in patients with mechanical heart valves [4], those with severely impaired renal function, or individuals with antiphospholipid syndrome [5].

OAT inevitably carries a risk of bleeding. The patient’s thromboembolic and bleeding risk must be constantly reassessed, maintaining the anticoagulation effect in a safe situation to minimize complications [6].

There is evidence that OAT is associated with adverse events due to its use, as well as prescription and administration errors [7]. One systematic review revealed that, specifically in the context of DOAC use, the proportion of patients encountering prescribing, dispensing, or administration errors across studies varied from 5.3% to 37.3%. The observed variability in error rates was frequently linked to the spectrum of indicated DOACs, and focused on either specific types of errors or a range of mistakes throughout the different stages of the medication use process [7].

Approximately 33% of emergency department visits for adverse drug events are related to the use of VKAs [8]. Patients who experience an anticoagulation-related adverse event during hospital admission have a high mortality rate within the first 30 days [9,10].

Owing to the specific characteristics of this group of drugs and the consequences that improper management can have, several multidisciplinary experiences are formed to reduce the events associated with OAT, intending to decrease adverse events through standardized, evidence-based practice [11,12].

Nurses are recognized as key healthcare professionals in ensuring high-quality care for patients receiving oral anticoagulant therapy (OAT) [13]. Within this context, health education represents a fundamental component of nursing practice. Several studies emphasize the importance of standard educational content, including basic principles of hemostasis, correct medication administration, and appropriate circumstances for contacting healthcare providers [14,15].

Beyond administering OAT and offering ongoing counseling on stroke and bleeding risks, nurses play a critical role in monitoring the International Normalized Ratio (INR) in patients receiving vitamin K antagonists (VKAs), interpreting these values, adjusting dosages accordingly, and implementing self-management and self-monitoring programs for patients on these medications [16].

Despite their critical role in oral anticoagulant therapy (OAT) management, observational studies have identified persistent knowledge gaps among nurses. These include uncertainties related to appropriate OAT dosing during thrombotic events [17] and safety considerations concerning the use of direct oral anticoagulants (DOACs) in elderly or oncology populations [18]. A survey by Ferguson (2016) further highlighted deficiencies in nurses’ understanding of drug–drug interactions, dietary and lifestyle influences on anticoagulant effectiveness, and clinical indications for DOAC use [16,19,20].

Evidence suggests that nurses’ knowledge of OAT improves following participation in targeted educational programs focused on this drug class [21,22,23,24,25]. In this context, self-efficacy has emerged as a key construct in nursing education and professional development. Defined as an individual’s confidence in their ability to perform specific tasks under defined conditions, self-efficacy is a central element of Bandura’s social cognitive theory [26,27]. It is a dynamic cognitive process shaped by four main sources: personal mastery (successful repetition of a task), vicarious experience (learning through observation), symbolic experience (verbal persuasion and peer encouragement), and emotional arousal (managing stress in performance situations) [27].

By mediating the relationship between knowledge and action, self-efficacy serves as a reliable proxy for assessing nursing competence in specific clinical contexts [28]. Higher self-efficacy among nurses is associated with higher quality patient care and better outcomes, as nurses who express higher levels of self-efficacy can spend more effort managing patient problems. It positively influences professional skills development and, therefore, affects whether a nurse is competent and professionally prepared for different clinical practice contexts [29,30,31]. In this context, studies have aimed to operationalize nurses’ self-efficacy in cancer treatment-induced cardiotoxicity [32], ostomy care [33], pressure ulcers [34], and oral anticoagulation management [35].

Nursing self-efficacy in oral anticoagulation management (SE-OAM) is the nurses’ belief in their ability to manage oral anticoagulant therapy. SE-OAM represents nurses’ confidence level in their ability to perform evidence-based tasks effectively for the optimal management of anticoagulated patients [35].

Regarding the background, we identified only one validated instrument that examines self-efficacy in the management of oral anticoagulant therapy among nurses. This questionnaire was developed by Magon et al. in Italy [35]. The Nursing Self-efficacy for Oral Anticoagulant Therapy Management (SE-OAM) questionnaire assesses five domains of anticoagulant management: clinical management, care management, education, care monitoring, and clinical monitoring. These domains evaluate the self-perceived efficacy of nurses in aspects such as using a blood coagulometer device, knowledge of clinical assessment tools, abilities such as identifying the most suitable patients for self-management formation, patient education, assessment of modifiable risk factors, the use of eHealth systems, and the identification of poor medication adherence and limited quality of life aspects [35]. Nevertheless, to the best of our knowledge, a scale to assess nursing self-efficacy in oral anticoagulated management is unavailable in our country.

This study centers on the content validity and cross-cultural adaptation of the Spanish version of the Nursing Self-Efficacy Scale for Oral Anticoagulation Management (SE-OAM-SV) among nurses in Spain. The adaptation process aims to ensure the scale’s cultural relevance, linguistic clarity, and practical applicability within the Spanish healthcare context. By validating this instrument, this study seeks to support the development of targeted educational interventions that enhance nurses’ competencies in managing oral anticoagulant therapy.

## 2. Materials and Methods

### 2.1. Design

A methodological design to develop the validity and reliability of the Spanish version of the SE-OAM was used in this study.

Different authors have carried out this methodology for the cross-cultural adaptation of instruments, with various modifications according to the purposes of their studies [36,37,38,39,40,41,42,43]. We used a methodology consisting of the following phases to obtain a version of the SE-OAM in Spanish:

### 2.2. Translation and Back-Translation

The original version in Italian [35] was translated into Spanish by two independent clinical nurses with advanced knowledge of Italian. These two translations were reviewed by a third nurse with extensive clinical experience and knowledge in the Italian language who worked with the researchers to develop a consensus version of SE-OAM in Spanish (SE-OAM-SV).

The consensus Spanish version of the SE-OAM-SV was back-translated to Italian by two university nurse professors with a high level of knowledge in Italian, who were experts in questionnaire validation. Thus, this back-translation process resulted in two new documents in Italian. This document was compared item by item with the original SE-OAM by a different translator to identify new discrepancies. The questions raised regarding the consensus translation and interpretability of the meaning of the items that raised questions of the back-translations versions were resolved by e-mail by the authors of the scale, who obtained a consensus SE-OAM-Spanish version (SE-OAM-SV).

### 2.3. Experts Review

A panel of experts in anticoagulation management in Spain evaluated the adequacy and relevance of each item in the SE-OAM-SV using a 5-point Likert scale, ranging from 1 (not suitable/not relevant) to 5 (very suitable/very relevant).

The expert panel was composed of nurses with at least two years of professional experience, all actively working in anticoagulation clinics within the Spanish healthcare system. This criterion ensured that the evaluators possessed practical expertise in the clinical context targeted by the instrument.

### 2.4. Content Validity Analysis

Following the methodology described by Polit and Beck [44] and other authors [38,41,43], the content validity of the questionnaire was analyzed by calculating the content validity index (CVI), modified kappa coefficient (k) and Aiken’s V value for each item and the global questionnaire, which was based on the ratings made by the group of experts, using the following equations [45]:

(a) Content validity index (CVI)
  CVI = number of experts who evaluated the item with 4 or 5 (A)/total of experts (N)
(b) Modified kappa (k)
  $$k=\frac{\left(I-CVI\right)-Pc}{1-Pc}$$

The I-CVI is the coefficient of internal validity, previously calculated for each item. In contrast, the Pc (probability of chance agreement) is the probability of chance agreement between observers and is calculated through the following formula:

$$Pc=\left[\frac{\left[N!\right]}{\left[A!\left(N-A\right)!\right]}\right]\times 0.5^{N}$$

(c) Aiken’s V. Its equation, algebraically modified by Penfield and Giacobbi (2004) [46], is as follows:
  $$V=\frac{X-l}{k}$$

X is the mean of the experts’ ratings, l is the lowest possible score, and k is the range of possible values of the Likert scale used. For example, if the lowest score is 1 and the highest score is 5, then k = 5 – 1 = 4. Once calculated, Aiken’s V confidence intervals were obtained using the scoring method [47] of the interval:

$$L=\frac{2nkV+z^{2}-z\sqrt{4nkV(1-V)+z^{2}}}{2(nk+z^{2})}$$

And for the upper limit of the interval:

$$U=\frac{2nkV+z^{2}+z\sqrt{4nkV(1-V)+z^{2}}}{2(nk+z^{2})}$$

L: lower limit of the interval; U: upper limit of the interval; z: value in standard normal distribution; V: Aiken’s V calculated by formula c; n: number of experts.

The CVI, modified kappa, and Aiken’s V were calculated with a database created in Excel 365, using the assessments of the expert group and according to their respective formulas.

### 2.5. Pilot Study

A descriptive cross-sectional study, developed across Spain, was designed to use the SE-OAM-SV for the first time and identify problems in its comprehension.

The Participants in this pilot study were also asked to reflect on any misunderstandings or difficulties within the SE-OAM-SV.

The population likely to participate in the pilot study was composed of RNs on active duty during December 2024 in different areas of care, from specialized to primary care in Spain. The objective was to collect 100 responses through purposive sampling.

### 2.6. Data Collection

Expert evaluation. The experts were contacted by phone, explaining the project and requesting their participation; once they agreed, the consensus translation of the SE-OAM-SV was sent to them, with instructions to evaluate the adequacy and comprehensibility of each item. They were also asked to propose better ways to express the items if they had any suggestions. The participation of this group was carried out voluntarily, anonymously, and confidentially using a questionnaire through the Microsoft Forms platform. The experts identified were asked to accept the Declaration of Consent if they were interested in participating in the study, according to the Data Protection Law in force in Spain.

Pilot Study. The pilot study was conducted via an ad hoc questionnaire distributed through social media and e-mail to nurses working in Spain via the Microsoft Forms platform. The participants were selected by convenience sampling. First, informed consent was requested, as the pilot project and voluntary participation were indicated through informed consent. All responses were collected anonymously, and no personally identifiable information was requested or stored, which complies with the European Union’s General Data Protection Regulation (GDPR) and the Spanish Organic Law 3/2018 on the Protection of Personal Data and Guarantee of Digital Rights. Microsoft Forms ensured secure data transmission and storage through encrypted channels, and access to the data was restricted to the research team via institutional credentials.

In the first section, sociodemographic and professional data were collected (age, sex, Autonomous Community, academic level, professional experience in years, possession of nursing specialty, time working in the anticoagulation clinic, specific training in the last 5 years, VKA dosing qualification, time titrating VKA doses). In the second part of the questionnaire, the SE-OAM-SV was attached. In addition to the questionnaire responses, they could identify problems with items’ comprehension, providing their comments.

The experts’ data were collected during October and November 2024, and the pilot study was conducted during December 2024. The participation of this group was carried out voluntarily, anonymously, and confidentially using a questionnaire through the Microsoft Forms platform.

Approval of the authors to adapt the original scale was also obtained by contacting them via e-mail.

### 2.7. Ethical Considerations

Arianna Magon, the original developer of the SE-OAM, granted permission for the use of the questionnaire in this study.

The study was conducted in accordance with the Declaration of Helsinki, and approved by the Committee for Human Studies of the Universitat de València with verification code SC7LE98ZAA12Z5Y1 on 17 July 2024.

The purpose and benefits of the research; written consent was obtained from all participants. Participant anonymity was guaranteed.

## 3. Results

### 3.1. Results of the Translation Process

Some aspects of the SE-OAM scale items required clarification during the translation process from Italian to Spanish. Specifically, in item 12, the term “modalità di assunzione’” was unclear in its translation to “métodos de ingesta”. The authors of the questionnaire were contacted, and the item was modified to “formas de administración”, as it referred to guidelines on how each anticoagulant, whether VKAs or DOACs, should be taken.

The results of the translation from Italian to Spanish are presented in Table 1.

### 3.2. Characteristics of Experts

Among 40 experts contacted by phone, 67.5% (27) responded to the questionnaire. Of these, 81.5% (22) were women. The mean age of the total participants was 47.1 years with a standard deviation (SD) of 9.58. The mean years of professional experience was 23.3 years as a nurse with an SD of 10.4. The mean years of experience managing anticoagulant treatment was 7.39 with an SD of 5.56. Regarding their academic degree, 11.1% (3) held a philosophical degree, 14.8% (4) had a master’s degree, and 74.1% (20) were graduates. Overwall, 85.2% (23) of the experts had specific training in anticoagulation therapy management. They came from ten autonomous communities: Aragón (*n* = 1); Andalucía (*n* = 6); Canary Islands (*n* = 1); Catalonia (*n* = 2); Valencian Community (*n* = 6); Madrid (*n* = 4); Galicia (*n* = 1); La Rioja (*n* = 1); Murcia (*n* = 4); and Basque Country (*n* = 1).

### 3.3. Content Validity Results

From the 27 expert evaluations, the CVI and Aiken’s V test values for each item were calculated. When analyzing the CVI, 28.57% (n = 6) did not reach the acceptable value of 0.78; however, among items 2, 4, and 16, their value were 0.741, which is very close to the 0.78 value. The expert study participants requested no additional clarifications. If we calculate the global CVI of the questionnaire (S-CVI), we see that it has a value of 0.86, greater than the value defined as acceptable.

Table 2 shows CVI and Aiken’s V values for each questionnaire item. 

Regarding the kappa index, we can appreciate that most items (85.71%) have an excellent level of concordance, while the rest have good concordance levels. Moreover, Aiken’s V results for each item were satisfactory, exceeding the acceptable value of 0.70 [48]. Therefore, we can say that the SE-OAM-SV presents adequate content validity, although the items with the lowest indexes should be improved to increase its content validity.

### 3.4. Pilot Study Results

A group of 100 nurses from all of Spain completed a questionnaire. The survey included questions about their socio-demographic background, academic degree, specific training on OAT, work field, and the roles performed concerning OAT, and the Spanish version of the Self-efficacy Oral Anticoagulation Management scale (SE-OAM-SV).

The pilot study participants requested no additional clarifications.

The average age was 41.3 years with a standard deviation of 11. The average years of professional experience was 17.3 years with an SD of 11. 79% were women, only one respondent preferred not to disclose their sex, and the rest were men. In total, 7% held doctoral degrees, followed by 34% with master’s degrees, and the rest were graduates. Most respondents did not have any nursing specialty. The scope of our participants’ work was hospitalization at 45%, primary and community care at 43%, anticoagulation clinic at 2%, and universities at 5% of the total. Only 28% of the participants had completed a specific course on OAT management; however, more than half of the sample, 64% of the total, reported performing INR checks in their workplace. Nevertheless, fewer than half, 46%, provide health education on OAT to patients receiving these treatments, and only 22% developed specific self-management programs with VKA.

Regarding the results obtained after completing the Spanish version of the SE-OAM questionnaire, the average score of the participants was 56.8 points. The results in each dimension of SE-OAM-SV are shown in Table 3.

### 3.5. Reliability Analysis

After the pilot test, using the responses from the participating nurses, Cronbach’s alpha was calculated. The Spanish version of the Se-OAM questionnaire demonstrated high internal consistency with a Cronbach’s alpha value of 0.96.

The Cronbach’s alpha did not subsequently increase to a significantly higher value when any of the items were removed, except for item one, where the value remained the same as the overall value.

Table 4 shows the item analysis of the Spanish version of the Nursing Self-Efficacy Scale for Oral Anticoagulation Management (SE-OAM).

Table 5 shows the dimension analysis of the Spanish version of the Nursing Self-Efficacy Scale for Oral Anticoagulation Management (SE-OAM).

## 4. Discussion

Self-efficacy is a critical mediator between knowledge and clinical practice, underscoring its influence on implementing specific professional competencies. Educational interventions deliberately designed to support the replication of effective behaviors, facilitate peer-recognized experiential learning, and foster a sense of competence—through both theoretical instruction and practical application—have been shown to enhance self-efficacy significantly [27].

The adoption of standardized nursing terminologies has advanced the conceptual clarity of diagnostic reasoning and the clinical interventions nurses perform. These frameworks have also emphasized the role of care complexity as an essential variable in guiding resource allocation, forecasting hospitalization costs, and supporting comprehensive care planning [49,50].

Measuring self-efficacy among nursing professionals has become valuable for assessing their confidence in performing specific tasks across diverse clinical environments. Despite the acknowledged role of Spanish nurses in managing oral anticoagulant therapy (OAT) within primary and specialized care settings, there remains a paucity of national studies evaluating nurses’ knowledge or self-efficacy related to these treatments.

To address this gap, the SE-OAM questionnaire, developed by Magon et al. [35], represents a validated instrument for assessing nurses’ self-efficacy in the management of OAT. However, this tool has not yet undergone cross-cultural adaptation beyond its original Italian context. Therefore, the present study aimed to adapt the SE-OAM questionnaire for use in Spanish and validate its application in a sample of Spanish nurses.

Regarding the content validity of the SE-OAM-SV, we observed that up to six items had a CVI below desirable values, although for three of them, it was very close to the established limits. Only item 12 was reformulated among the six items to enhance its content validity and comprehensibility. Nonetheless, it is essential to note that this questionnaire exhibits an overall Content Validity Index (CVI) deemed acceptable according to the criteria established in the scientific literature. In our case, the global CVI is 0.86, which is lower than the 0.94 value obtained in the original version, but achieves satisfactory levels in the reliability of the SE-OAM-SV. Furthermore, two additional tests were conducted to evaluate content validity, the Kappa index, and Aiken’s V, which reaffirmed the quality of the content validity with values of 0.855 and 0.850, respectively.

The Cronbach’s index was calculated to study the internal consistency of the SE-OAM-SV. The current version of the SE-OAM questionnaire had demonstrated satisfactory results (Cronbach’s Alpha = 0.96), with results very similar to those of the Italian version (Cronbach’s Alpha = 0.951).

The 21 items composing the original scale [35] have been retained. In the SE-OAM-SV scale, it was observed that Cronbach’s alpha did not rise to a significantly higher value upon removing any of the items, except for item one, where the value remained the same overall.

Focusing on the values obtained for internal consistency for each of the domains that are part of the SE-OAM, all the values of our domains were excellent, exceeding the score of 0.90 for all of them. The Cronbach’s alpha values from our study compared with those obtained in Magon’s work, with two determinations, which are, respectively: Clinical Management (0.95 vs. 0.85 vs. 0.89), Care Management (0.94 vs. 0.84 vs. 0.85), Clinical Monitoring (0.96 vs. 0.90 vs. 0.90), Education (0.93 vs. 0.93 vs. 0.93), and Care Monitoring (0.94 vs. 0.96 vs. 0.95). If we compare our data with those obtained in the two analyses conducted in Magon et al.’s work (Magon et al., 2021), our data indicate higher scores in three of the five domains that make up the SE-OAM compared with Italian study [35], with the values of the other two dimensions being very similar between both studies.

The standardized mean score of our participants on the SE-OAM scale was 56.8 (SD = 20.9) out of a maximum of 100, which is notably higher than the score reported in the original Italian validation study (56.8 [20.9] vs. 42.97 [15.46]). Across individual domains, our participants consistently achieved higher mean scores than those in the Italian sample, except for the ‘Clinical Monitoring’ domain.

In the ‘Clinical Management’ domain, our sample scored 61.8 (SD = 22.1), compared to 56.73 (SD = 15.85) and 55.51 (SD = 15.97) in the Italian study. This domain assesses nurses’ confidence in understanding clinical indications for oral anticoagulant therapy (OAT), interpreting coagulation parameters such as PT-INR, and recognizing and managing thromboembolic or hemorrhagic complications—skills critical to ensuring safe and effective care delivery.

In the ‘Care Management’ domain, our participants scored 49.4 (SD = 22.9), substantially higher than the Italian scores of 31.61 (SD = 19.44) and 31.65 (SD = 19.73). This domain evaluates confidence in the use of portable coagulometers, application of clinical risk stratification tools (e.g., CHA_2_DS_2_-VASc, HAS-BLED), and identification of patients suitable for self-monitoring or self-management of vitamin K antagonists (VKAs).

For the ‘Education’ domain, our participants reported a mean score of 57.5 (SD = 21.9), compared to 44.67 (SD = 18.62) and 43.76 (SD = 17.56) in the Italian sample. This domain reflects nurses’ confidence in delivering patient education based on evidence-based guidelines and tailoring interventions according to individual health determinants.

The most significant relative difference was observed in the ‘Care Monitoring’ domain, with a mean score of 66.6 (SD = 24.9) in our sample versus 30.72 (SD = 22.73) and 30.51 (SD = 22.34) in the Italian study. This domain encompasses confidence in modifying modifiable factors such as patient knowledge, health literacy, and quality of life.

The only domain in which our participants scored lower was ‘Clinical Monitoring’, with a mean of 48.1 (SD = 28.6), compared to 51.27 (SD = 19.03) and 49.49 (SD = 20.57) in the Italian sample. This domain assesses the use of eHealth tools for monitoring anticoagulation control quality.

We have not identified any studies directly associating practical competencies in OAT management with SE-OAM scores, highlighting an important gap in the current literature that warrants further investigation.

It is noteworthy that the average age of our participants was higher than that in the Italian study, 41.3 (17.3) vs. 37.5 (10.59) vs. 36.85 (10.15) median years. Similarly, we must highlight that the level of years of professional experience is also higher in our study compared to that of Magon et al. (17.3 (11) vs. 14.29 (11.27) vs. 12.90 (10.66)). This finding is consistent with other studies on nursing self-efficacy, which indicate that older age and more years of professional practice are associated with higher levels of self-efficacy [51,52].

Nearly half of our sample works in primary care compared with the Italian study, where participants primarily work in the hospital setting. With the introduction of portable coagulometers, Spain has shifted from a centralized model of managing vitamin K antagonists (VKAs) within hospital-based anticoagulation clinics to a decentralized approach in primary care health centers. The routine management of oral anticoagulant therapy (OAT) by primary care nurses in Spain involves conducting INR checks and monitoring patients on VKAs, supported by standardized protocols from anticoagulation clinics. This routine practice may account for the higher levels of self-efficacy observed in our study compared to the findings of Magon et al., where the sample was predominantly hospital-based.

Although the self-efficacy results are better than those of the Italian study, these data indicate a wide range of improvements in all aspects of the SE-OAM. This finding aligns with studies that already report a lack of knowledge in managing oral anticoagulant therapy and antithrombotic prophylaxis measures [16,17,19,20,51,53]. Evidence indicates the influence of the level of knowledge on the self-efficacy expressed by nurses in performing specific tasks [51,54,55].

Further research is needed to establish more definitive associations between nurses’ self-efficacy in managing oral anticoagulant therapy and clinical outcomes such as patient safety, morbidity, mortality, and the overall quality of anticoagulation management. Generating such evidence would be pivotal for informing curriculum development and enhancing clinical practice, particularly in designing and implementing specialized training programs for nurses involved in oral anticoagulant therapy management.

Regarding the study’s limitations, the internal consistency analysis was conducted through a pilot test involving a small sample of Spanish nurses. Therefore, further studies with more diverse samples are necessary to more robustly evaluate the SE-OAM-SV’s reliability and generalize its findings to the broader population of nurses in Spain.

Although the original version of this questionnaire has been available since 2021, its use has been limited. To our knowledge, no studies have assessed the SE-OAM outside the original validation context, underscoring the need for further research investigating nurses’ self-efficacy in managing oral anticoagulant therapy globally.

The cross-cultural adaptation of the SE-OAM to Spanish impacts the study of nurses’ self-efficacy in managing oral anticoagulant treatments in Spanish-speaking communities. This validated tool can be used to study the relationship between nurses’ self-efficacy and the clinical outcomes of anticoagulated patients resulting from their optimal management.

Additionally, having a tool that measures the level of self-efficacy in the management of oral anticoagulant therapy (OAT) will allow us to study its relationship with the level of specific knowledge about OAT and the development of clinical skills necessary for proper management, based on standardized procedures for the development of evidence-based practice.

As future lines of research, we aim to analyze the psychometric properties of the SE-OAM-SV in Spain by conducting a factor analysis with a larger sample to establish the factorial structure of the SE-OAM-SV and to re-evaluate its construct validity.

## 5. Conclusions

The results of this research support the reliability and validity of the SE-OAM-SV for its use among Spanish nurses to measure their level of self-efficacy in oral anticoagulation management.

The availability of a Spanish version of the SE-OAM supports the execution of larger-scale studies that compare self-efficacy levels in OAT across different contexts and validate the tool in other Spanish-speaking countries.

## Figures and Tables

**Table 1:** Spanish version of SE-OAM (SE-OAM-SV).

Item Number	SE-OAM-SV Item
Item 1	ITA: Comprendere l’indicazione clinica al trattamento terapeutico SPA: Comprender la indicación clínica del tratamiento terapéutico. ENG: To understand the clinical indication for the treatment
Item 2	ITA: Interpretare i valori dei test standardizzati di laboratorio (ad es. PT-INR; funzionalità epatica e renale; D-dimero; aPTT) in relazione al potenziale rischio clinico della persona assistita SPA: Interpretar los valores de las pruebas de laboratorio estandarizadas (por ejemplo, PT-INR; función hepática y renal; dímero D; aPTT) en relación con el riesgo clínico potencial del paciente. ENG: To interpret the anticoagulation parameters (e.g., PT-INR, D-dimer) related to patients’ clinical risks
Item 3	ITA: Utilizzare ed interpretare i parametri dei Point Of Care (POCTs) (ovvero coagulometri portatili) per la valutazione dell’assetto coagulativo in condizioni di emergenza/urgenza o nell’utilizzo del monitoraggio cronico della TAO con cumarinici SPA: Utilizar e interpretar los parametros de los Point Of Care (POCTs) (es decir, coagulómetros portátiles) para la valoración de la coagulación en condiciones de emergencia/urgencia o en la utilizacion de la monitorización crónica de la terapia anticoagulante oral con cumarínicos. ENG: To use the blood coagulometer device (i.e., Point of care, POCT) for assessing and monitoring the coagulation profile in acute or chronic condition
Item 4	ITA: Interpretare i valori delle scale di misurazione del rischio clinico (ad es, CHA_2_DS_2_-VASc Score; HAS-BLED Score) SPA: Interpretar los valores de las escalas de medida del riesgo clínico (por ejemplo, CHA_2_DS_2_-VASc Score; HAS-BLED Score) ENG: To interpret the results of clinical assessment tools (e.g., CHA_2_DS_2_-VASc Score; HAS-BLED) related to patients’ clinical risks
Item 5	ITA: Interpretare i segni e sintomi delle principali complicanze acute/croniche della TAO SPA: Interpretar los signos y síntomas de las principales complicaciones agudas/crónicas de la TAO ENG: To interpret the main signs and symptoms of oral anticoagulation therapy complications (i.e., thromboembolic or hemorrhagic)
Item 6	ITA: Gestire i segni e sintomi delle principali complicanze acute/croniche della TAO SPA: Manejar los signos y síntomas de las principales complicaciones agudas/crónicas de la TAO ENG: To manage the main signs and symptoms of oral anticoagulation therapy complications (i.e., thromboembolic or hemorrhagic)
Item 7	ITA: Identificare i pazienti idonei a modelli di autogestione della TAO (ossia modelli di gestione Self-Monitoring, SM) SPA: Identificar a los pacientes adecuados a los modelos de autogestión de la terapia anticoagulante (es decir, modelos de autoanálisis, autocontrol) ENG: To identify the eligible patients for OAC self-management (i.e., Self-Monitoring, SM)
Item 8	ITA: Condividere e programmare la gestione della TAO in un sistema integrato di cure con i Centri Emostati e Trombosi (CET) (o altra struttura ospedaliera/territoriale in cui è in carico l’assistito) ed equipe multidisciplinare SPA: Compartir y planificar la gestión de la TAO en un sistema de atención integrado con los Centros de Hemostasia y Trombosis (CET) (u otra estructura hospitalaria/territorial en la que el paciente es asistido) y equipos multidisciplinares. ENG: To plan and share the OAC management paths considering the characteristics of the healthcare systems (e.g., Anticoagulation Clinics) and using a multidisciplinary approach
Item 9	ITA: Ricercare e comprendere nuove evidenze cliniche/raccomandazioni nella gestione ottimale della TAO SPA: Investigar y comprender nuevas evidencias/recomendaciones clínicas en el manejo óptimo de la TAO ENG: To research and apply the best evidence/guidelines in the OAC management
Item 10	ITA: Lavorare secondo protocolli e algoritmi condivisi nella gestione del dosaggio terapeutico per i farmaci cumarinici SPA: Trabajar según protocolos y algoritmos compartidos en el manejo de dosis terapéuticas de cumarínicos. ENG: To work considering protocols and shared algorithms for managing the therapeutic dosage for Antagonist Vitamin K
Item 11	ITA: Identificare, oggettivare e valutare la comprensione, le conoscenze, abitudini/stili di vita (ossia alimentazione, attività fisica, etc) motivazione e credenze dell’assistito rispetto alla TAO SPA: Identificar, objetivar y evaluar la comprensión, el conocimiento, los hábitos/estilos de vida (es decir, nutrición, actividad física, etc.), la motivación y las creencias del paciente con respecto a la TAO. ENG: To objectively measure knowledge, lifestyle habits (e.g., nutrition, physical activity), motivation, and patients’ beliefs about OAC
Item 12	ITA: Progettare interventi educativi efficaci per l’assistito e/o caregiver rispetto alla condizione clinica e trattamento terapeutico (ossia, funzionalità, monitoraggio, possibili complicanze ed interazioni, modalità di assunzione) SPA: Diseñar intervenciones educativas efectivas para el paciente y/o cuidador con respecto a la condición clínica y el tratamiento terapéutico (es decir, funcionalidad, seguimiento, posibles complicaciones e interacciones, forma de administración) ENG: To plan effectiveness educational interventions for patients and their caregivers, considering their clinical condition and treatment
Item 13	ITA: Pianificare ed attuare metodologie educative specifiche (ovvero di Self-Monitoring, Case-Management, gruppi di formazione tra pari) finalizzati al raggiungimento di competenze teoriche ed abilità tecniche dell’assistito nella gestione autonoma della TAO SPA: Planificar e implementar metodologías educativas específicas (es decir, autoanálisis (Self-Monitoring), gestión de casos, grupos de capacitación entre pares) destinadas a lograr habilidades teóricas y técnicas del paciente en el manejo autónomo de TAO ENG: To plan and implement specific educational approaches (i.e., self-monitoring, Case Management, peer training groups) aimed at achieving better patients’ skills in self-management of OAC
Item 14	ITA: Personalizzare l’educazione alla gestione della TAO in casi clinici complessi (ad es, stato di gravidanza, oncologico, epatopatia, nefropatia o emodialisi) SPA: Personalizar la educación sobre el manejo de la TAO en casos clínicos complejos (p. ej. embarazo, oncológico, enfermedad hepática, nefropatía o hemodiálisis) ENG: To customize the education for OAC management, considering complex clinical cases (e.g., pregnancy, oncology, liver disease, nephropathy, or hemodialysis)
Item 15	ITA: Identificare le esigenze educative individuali (singola persona) SPA: Identificar necesidades educativas individuales (una sola persona) ENG: To identify the individual educational needs
Item 16	ITA: Identificare le esigenze di comunità (ad es, in relazione ad un territorio specifico che usufruisce ad un unico centro ambulatoriale) SPA: Identificar las necesidades educativas de la comunidad (por ejemplo, en relación con un territorio específico que utiliza un único centro ambulatorio) ENG: To identify the community educational needs
Item 17	ITA: Indirizzare l’assistito nella gestione tecnico-amministrativa del trattamento terapeutico (ad es, richiesta di esenzione, modalità di prenotazione appuntamenti, etc) secondo disposizioni regionali specifiche e/o nazionali relative alla tutela della cronicità SPA: Dirigir al paciente en la gestión técnico-administrativa del tratamiento terapéutico (p. ej., solicitud de dispensa, forma de concertación de citas, etc.) según disposiciones específicas autonómicas y/o nacionales relativas a la protección de la cronicidad ENG: To address the patient who requires OAC to proper healthcare service in accordance with the domestic regulations
Item 18	ITA: Conoscere sistemi di telemedicina (e-health) per il monitoraggio della qualità terapeutica (esempio: TTR) e pianificazione dei controlli successivi SPA: Conocer los sistemas de telemedicina (e-health) para la monitorización de la calidad terapéutica (ejemplo: TTR) y la planificar los controles posteriores ENG: To know telemedicine systems (e-health) for monitoring the quality of anticoagulation control (i.e., Time in Therapeutic Range, TTR) and for planning subsequent follow-ups
Item 19	ITA: Utilizzare sistemi di telemedicina (e-health) per il monitoraggio della qualità terapeutica (esempio: TTR) e pianificazione dei controlli successivi SPA: Utilizar sistemas de telemedicina (e-health) para la monitorización de la calidad terapéutica (p.e.; TTR) y planificar los sucesivos controles ENG: To use telemedicine systems (e-health) for monitoring the quality of anticoagulation control (i.e., Time in Therapeutic Range, TTR) and for planning subsequent follow-ups
Item 20	ITA: Identificare e correggere fattori di rischio modificabili (ad es, inadeguati stili di vita, conoscenze limitate, bassa motivazione) nella promozione dell’aderenza terapeutica SPA: Identificar y corregir factores de riesgo modificables (p. ej., estilo de vida inadecuado, conocimientos limitados, baja motivación) para promover la adherencia terapéutica. ENG: To identify and correct the modifiable risk factors (i.e., inadequate lifestyles, limited knowledge, low motivation) for promoting an adequate treatment adherence
Item 21	ITA: Valutare l’aderenza terapeutica e qualità di vita dell’assistito in TAO SPA: Evaluar la adherencia terapéutica y calidad de vida del paciente en TAO ENG: To assess treatment adherence and quality of life of patients treated with OAC

OAC: oral anticoagulant therapy. The English version of the questionnaire is a non-validated translation of the one developed by Magon et al., 2021 [35].

**Table 2:** The content validity index (CVI), Kappa index (K), and Aiken’s V for each item of the Spanish version of Se-OAM (SE-OAM-SV).

Item Nº	CVI	K	Aiken’s V	IC 95%
1	0.96	0.96	0.93	(0.87–0.97)
2	0.74	0.74	0.81	(0.73–0.88)
3	0.93	0.93	0.91	(0.85–0.96)
4	0.74	0.74	0.76	(0.67–0.83)
5	1.00	1.00	0.94	(0.88–0.97)
6	0.85	0.85	0.81	(0.73–0.88)
7	0.78	0.78	0.75	(0.66–0.88)
8	0.67	0.65	0.78	(0.67–0.83)
9	0.89	0.89	0.86	(0.79–0.92)
10	1.00	1.00	0.92	(0.86–0.96)
11	0.93	0.96	0.91	(0.84–0.95)
12	0.96	0.96	0.88	(0.80–0.93)
13	0.92	0.93	0.89	(0.82–0.93)
14	0.81	0.81	0.81	(0.72–0.87)
15	0.93	0.93	0.92	(0.85–0.96)
16	0.74	0.74	0.77	(0.68–0.84)
17	0.89	0.89	0.87	(0.79–0.92)
18	0.67	0.65	0.74	(0.65–0.81)
19	0.63	0.60	0.70	(0.61–0.78)
20	0.96	0.96	0.93	(0.86–0.96)
21	0.96	1.00	0.95	(0.90–0.98)
GLOBAL	0.86	0.85	0.85	(0.61–0.98)

The content validity index (CVI), Kappa index (K), Aiken’s V Confidence Interval (IC).

**Table 3:** Standardized scores of the ratings obtained from the dimensions of the SE-OAM-SV scale.

	Clinical Management	Care Management	Education	Care Monitoring	Clinical Monitoring	Total
N	100	100	100	100	100	100
Mean	61.8	49.4	57.5	66.6	48.1	56.8
SD	22.1	22.9	21.9	24.9	28.6	20.9
Min	6.25	0.00	0.00	0.00	0.00	2.38
Max	100	100	100	100	100	100

N = total number of participants; SD = standard deviation; Min = minimum; Max = maximum.

**Table 4:** Item reliability analysis of the Spanish version of the Nursing Self-Efficacy Scale for Oral Anticoagulation Management (SE-OAM-SV).

Item Nº	Correlation Coefficient: Item-Total Score	Cronbach’s Alpha If Item Deleted
1	0.531	0.960
2	0.631	0.959
3	0.634	0.959
4	0.646	0.959
5	0.679	0.958
6	0.684	0.958
7	0.706	0.958
8	0.658	0.959
9	0.737	0.958
10	0.804	0.957
11	0.805	0.957
12	0.824	0.957
13	0.852	0.956
14	0.667	0.959
15	0.746	0.958
16	0.730	0.958
17	0.824	0.957
18	0.717	0.958
19	0.717	0.958
20	0.758	0.958
21	0.704	0.958
GLOBAL	0.531	0.960

**Table 5:** Dimension analysis of the Spanish version of the Nursing Self-Efficacy Scale for Oral Anticoagulation Management (SE-OAM).

	Correlation Coefficient: Item-Total Score	Cronbach’s Alpha If Item Deleted
Clinical management	0.77	0.95
Care management	0.87	0.94
Education	0.92	0.93
Care monitoring	0.83	0.94
Clinical monitoring	0.76	0.96

## Data Availability

The data that support the findings of this study are available from the corresponding authors upon reasonable request.

## Abbreviations

The following abbreviations are used in this manuscript:

CVI	Content Validity Index
DOACs	Direct-Acting Oral Anticoagulant
IC	Confidence Interval
INR	International Normalized Ratio
OAT	Oral Anticoagulant Therapy
SD	Standard Deviation
SE-OAM	Nursing Self-Efficacy for Oral Anticoagulant Therapy Management
SE-OAM-SV	Spanish version of the SE-OAM questionnaire
VKAs	Vitamin K Antagonists
